# Biomarkers of Oxidative Stress and Personalized Treatment of Pulmonary Tuberculosis: Emerging Role of Gamma-Glutamyltransferase

**DOI:** 10.1155/2012/465634

**Published:** 2012-05-07

**Authors:** Etienne Mokondjimobe, Benjamin Longo-Mbenza, Jean Akiana, Ulrich Oswald Ndalla, Regis Dossou-Yovo, Joseph Mboussa, Henri-Joseph Parra

**Affiliations:** ^1^Faculty of Health Sciences, Anti-Tuberculosis Centre, National Laboratory of Public Health, Marien Ngouabi University, Brazzaville, Democratic Republic of Congo; ^2^Faculty of Health Sciences, Walter Sisulu University, Private Bag X1, Eastern Cape, Mthatha 5117, South Africa

## Abstract

*Background*. The objectives were (i) to evaluate the impact of acute pulmonary tuberculosis (PTB) and anti-TB therapy on the relationship between AST, ALT, and GGT levels in absence of conditions related to hepatotoxicity; (ii) to evaluate the rate and the time of alterations of AST, ALT, and GGT. *Design and Methods*. A prospective followup of 40 adults (21 males; mean age of 34.7 ± 5.8 years) with active PTB on initial phase and continuation phase anti-TB. *Results*. Only 3% (*n* = 1) developed a transient and benign ADR at day 30 without interruption of anti-TB treatment. Within normal ranges, GGT decreased significantly from day 0 to day 60, while AST and ALT increased significantly and respectively. During day 0–day 60, there was a significant, negative, and independent association between GGT and AST. *Conclusion*. The initial two months led to significant improvement of oxidative stress. Values of oxidative markers in normal ranges might predict low rate of ADR.

## 1. Background

The concept of biomarkers is very important in this paper. The role of biomarkers is now exponentially increasing in guiding decisions in drug development and personalized medicine. A biomarker is not predictive or casual to a disease, but it can predict patients' response to compound by identifying certain patient groups that are more likely to response to the drug therapy or to avoid specific adverse events [1].

In patients with pulmonary tuberculosis (PTB), a significant improvement in oxidative stress and suppression of inflammatory response have been recently reported after the initial two-month therapy [2]. However, the literature showed that even after six months of successful chemotherapy, PTB is still associated with increased levels of circulating lipid peroxides and low plasma concentrations of antioxidants such as vitamin E [2, 3].

Before chemotherapy, Mycobacteria induce reactive oxygen species (ROS) production by activating both mononuclear and polymorphonuclear phagocytes. Tuberculosis is, therefore, characterized by poor antioxidants defence that exposes to oxidative host tissue damage [3, 4]. Yasuda et al. found that more than 50% of 113 PTB cases with normal liver function on admission showed abnormalities of transaminases that were aggravated up to 4th week after administration of drugs, and 80% up to the 8th week [5].

Almost 33% of the world population infected with *Mycobacterium tuberculosis* live in developing countries including Brazzaville, the Republic of Congo [6]. Anti-PTB drug-related adverse reactions [7] include benign or fatal hepatic transaminase elevation without any intervention [8, 9]. World Health Organization (WHO) established a major strategy of directly observed treatment short course (DOTS), which is adopted by the Republic of Congo, our country. The potentially hepatotoxic anti-TB drugs from the first-line regimen are isoniazide, rifampicin, and pyrazinamide [10].

The following conditions increase the risk of anti-TB drug-induced hepatitis: malnutrition, excessive alcohol intake, aging, chronic hepatic diseases including viral infections (HBV, HCV, and HIV/AIDS), female sex, ethnicity (Asians), concurrent administration of enzyme inducers, and inadequate compliance [7, 9, 11, 12].

### 1.1. Rationale

There are no data related to the chronological observation of liver enzymes and adverse drug reactions (ADR) in PTB patients. This information is useful to determine whether or not the antituberculosis drugs should be discontinued when hepatic dysfunction occurs. Indeed, Yasuda et al. showed that cases with higher peak values and exacerbation in transaminases had a tendency-delayed normalization [5].

With the current and increasing interest in oxidative stress [3], emphasis is to develop functional biomarkers of oxidative stress with epidemiological and clinical implications such as gamma-glutamyltransferase (GGT) [13–15] and alanine aminotransferase (ALT) [15], except for aspartate aminotransferase (AST) [14]. Therefore, the first objective of this study was to evaluate the impact of acute pulmonary tuberculosis (PTB) and anti-TB therapy on the relationship between AST, ALT and GGT levels in absence of conditions related to hepatotoxicity. The second objective of the study was to evaluate the rate and the time, pattern of alterations in AST, ALT, and GGT associated with ADR in these personalized conditions. The findings from this analysis of clinical courses of PTB patients will help the Anti-TB Centre to establish preventive measures for elevated liver enzymes and the background of oxidative stress activity anti-TB therapy.

## 2. Materials and Methods

### 2.1. Study Design

This study was approved by the local Research Ethics committee of the Faculty of Health Sciences of the Marien Ngouabi University of Brazzaville, Republic of Congo. The study was a followup prospectively undertaken according to the Helsinki Declaration after verbal consent was obtained from adult patients referred to the Anti-TB Centre in Brazzaville, Republic of Congo, Central Africa, between September 2003 and September 2004.

Inclusion criteria into the study included typical symptoms and signs of active PTB: chronic cough, chronic fever, sweating, fibrocavitary lung infiltrate on chest radiograph, and at least one sputum specimen staining positive Ziehl-Neelsen for acid fast bacilli. Diagnosis of PTB was based on the WHO criteria including a positive culture for *Mycobacterium tuberculosis* or negative culture associated with clinical and radiological features and response to treatment consistent with TB or histological studies [16].

Exclusion criteria were age <18 years old, age >50 years old, the conditions related to the high risk of anti-TB drug-induced hepatitis [7, 9, 11, 12], cigarette smoking, history of drug usage, blood transfusion previous, treatment for PTB, coexisting lung pathology, renal failure, diabetes mellitus, suspected multidrug resistant (MDR) and extensively drug resistant (XDR) TB, and additional criteria for women being pregnant or lactating, or females being menstruating at the time of blood collection.

Based on WHO recommended standard [16], patients received a regimen of isoniazid, rifampicin, pyrazinamide, and ethambutol for initial phase, followed by isoniazid and rifampicin at continuation phase. Approximately 10 mL of venous blood was drawn using disposable plastic syringes from PTB patients prior to initiation of anti-TB therapy (time 0 day) and at the ends of first month (time 30 days) and the second month (time 60 days) of the initial phase, and transferred immediately into disposable plain tubes.

### 2.2. Laboratory Analyses

Venous blood was also drawn at the end of the continuation phase, and at 15 days and 45 days after the end of the continuation phase. The serum was separated after centrifugation of the blood and kept frozen at −20°C before being analyzed at the National Laboratory of Public Health in Brazzaville, Republic of Congo.

Serum AST was evaluated by a kinetic determination. Malate dehydrogenase catalyzes the reaction of oxaloacetic acid with *β*-NADH_2_ by forming lactic acid and *β*-NAD. Serum ALT was evaluated by a kinetic determination. Lactate dehydrogenase catalyzes the reaction of pyruvic acid with *β*-NADH_2_ by forming lactic acid and *β*-NAD. Serum GGT was evaluated by an enzymatic colorimetric method. All laboratory measurements were performed using Biomé rieux reagents and automatic analyzer (Visual, Biomé rieux, Marcy l'Etoile, France).

The interassay coefficients of variation of these laboratory measurements were as follows: AST: 0.8%; ALT: 0.5%; GGT: 0.6%. The intraassay coefficients of variation were within the 0.87–2.1% interval.

### 2.3. Monitoring

Also the participants were followed clinically during anti-TB therapy course. Noxious or unintended response to a drug which occurs at doses often used in human beings [16] was reported.

### 2.4. Statistical Analysis

The data of the study were expressed as mean ± standard deviation for continuous variables and as proportions (%) for categorical variables.

One-way analysis of variance (ANOVA) with Bonferroni post hoc test for multiple comparisons was used to compare AST, ALT, and GGT means across the periods of the initiation phase (day 0, day 30, and day 60) and those of the continuation phase (at the end of treatment, 15 days and 45 days after the end of the continuation phase). Students *t*-test served to compare the means of liver enzymes before anti-TB treatment and the average of their means after the end of the global treatment. Linear-by-linear association with *P* for trend served to compare the proportions of ADR across the quintiles of AST, ALT, and GGT.

Simple coefficients “*r*” were determined between the liver enzymes, while the multiple linear regression was fitted with GGT as dependent variable and Age, AST, and ALT as independent variables for the mean values from the baseline and the end of the initial phase, at the end of 30 days (1 month) and 60 days (2 months) after the ignition of the anti-TB therapy. The assumptions were tested by the residual analysis (difference between the actual and the predicted score): histogram of residuals dependent variable residuals against independent variables, and normal probability. A *P* value < 0.05 was considered to be statistically significant. SPSS for Windows version 19.0 (SPSS Inc., Chicago, IL, USA) was used for all analyses of data.

## 3. Results

### 3.1. Characteristics of the Study Population

The study sample comprised of 40 patients (52.5%  *n* = 21 were males, and 47.5%  *n* = 19 were females with sex ratio almost 1 man: 1 woman and aged 34.7 ± 5.8 years).

### 3.2. ADR

At the day 30 after starting the initial phase, 1 patient (3%) experienced pruritus due to self-administration of 2 tablets of cotrimoxazole. His transient mild hepatic dysfunction defined by a peak of serum enzymes (AST, ALT, and GGT greater than two times of the upper limit of their normal range: AST < 26 UI/L, ALT < UI/L, and GGT = 7–34 UI/L), vomiting, diarrhoea, but without jaundice. Because normalisation of the liver enzymes and the relief of pruritus and gastrointestinal symptoms occurred after two-day antihistaminic treatment, the anti-TB drugs were not stopped. However, the day 30 liver enzymes values of this patient were not considered in the all statistical analyses.

### 3.3. Variations of Liver Enzymes

The mean values of AST (19.5 ± 8.6 UI/L), ALT (7.6 ± 3.2 UI/L), and GGT (20.4 ± 16.8 UI/L) before anti-TB therapy were not different (ANOVA, *P* > 0.05) from those observed at the end of the continuation phase (AST = 19.4 ± 8.5 UI/L; ALT = 7.5 ± 3.5 UI/L; GGT = 20.3 ± 16.8 UI/L), at day 15 after the end of the continuation phase (AST = 19.2 ± 5.8 UI/L; ALT = 9.3 ± 4 UI/L; GGT = 19.7 ± 12.1 UI/L), and at day 45 after the end continuation phase (AST = 19.5 ± 7.3 UI/L; ALT = 11.6 ± 4.7 UI/L; GGT = 21.6 ± 17.7 UI/L).

**Figure 1:**
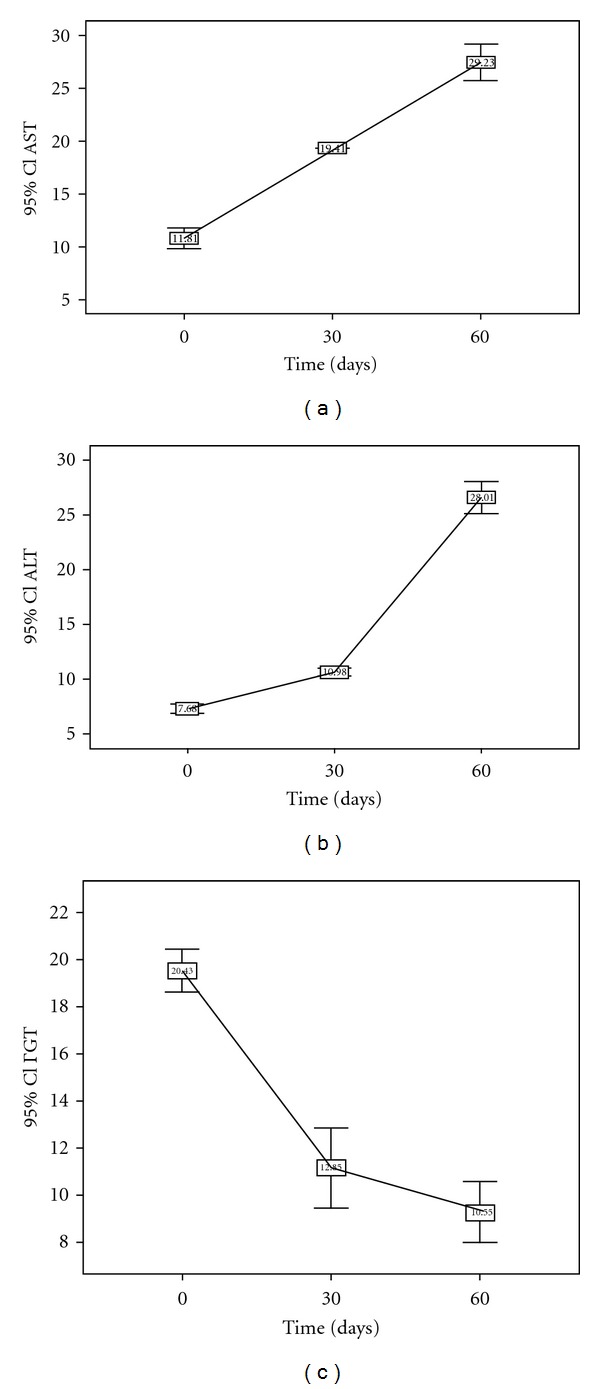
Variations of AST, ALT, and GGT during the two months of initial phase with their Bonferroni post hoc tests. *Day 0 versus day 30: *P* < 0.0001; day 0 versus day 60: *P* < 0.0001. *Day 30 versus day 60: *P* < 0.0001.

However, during the two months of the initial phase of anti-TB therapy, values of AST (SD: 3.1 VI/L day 0; 0.1 UI/L day 30; 5.4 UI/L day 60) increased significantly (ANOVA, *P* < 0.0001) across the time, while GGT values (SD: 2.8 UI/L day 0; 5.3 UI/L day 30; 4 UI/L day 60) decreased significantly across the time (ANOVA, *P* < 0.0001), respectively, (Figure 1).

**Figure 2:**
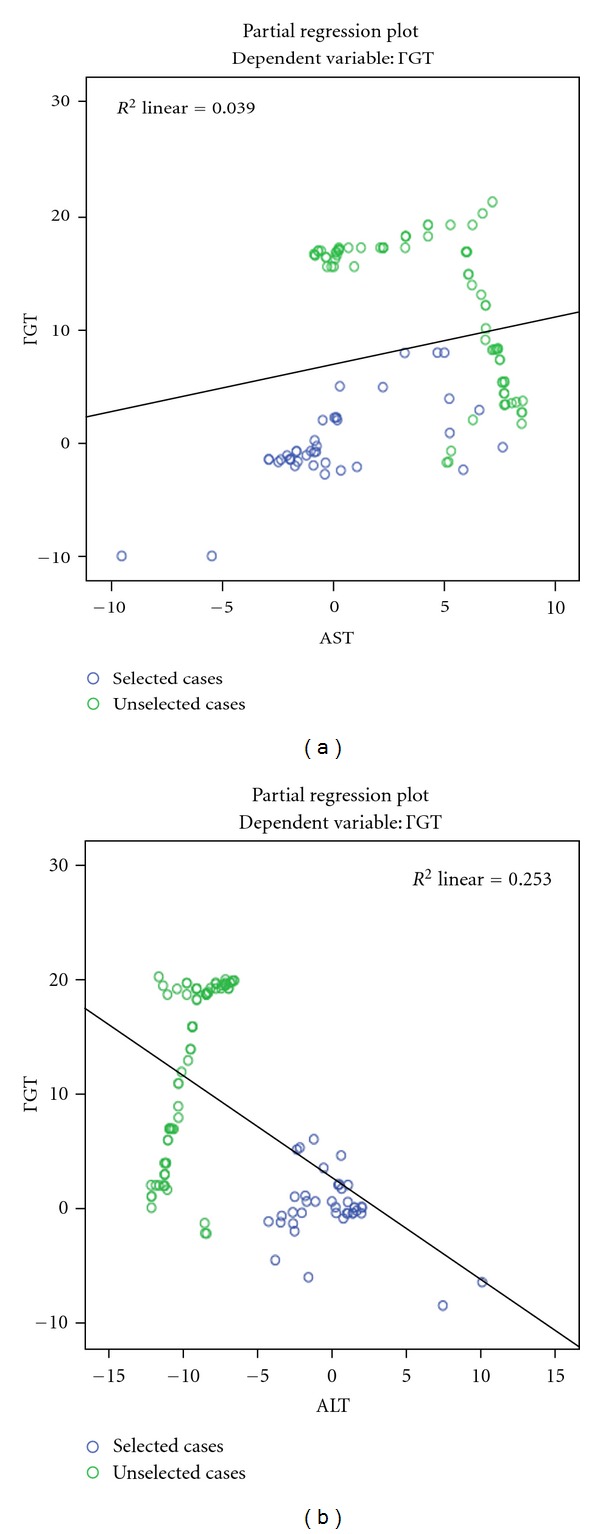
Multivariate relation between AST (a), ALT (b), and GGT at day 60 of initial phase of DOTS.

At each time of the initial phase of anti-TB therapy, a significant and positive correlation between liver enzymes was observed (Table 1). The level and the strength of association between GGT and AST was increasing with anti-TB therapy duration. The highest level of association between GGT and ALT, and that between AST and ALT, was observed at day 30 after the starting of the initial phase. During the initial phase of anti-TB therapy and after adjusting for confounding factors (age, sex, either AST or ALT) and identifying the independent determinants of GGT at each assessment time, there was a significant linear relationship between adjusted *R* square defining the variations of GGT and the different times of assessment (Table 2). At day 0 and day 30 after anti-TB treatment, only increasing ALT was the significant explanatory variable of the variations of GGT. At the day 60 after the anti-Tb therapy, 52% of variations in concentrations of GGT were significantly and independently explained by the increase in AST and the decrease in ALT levels (Figure 2).

**Figure 3:**
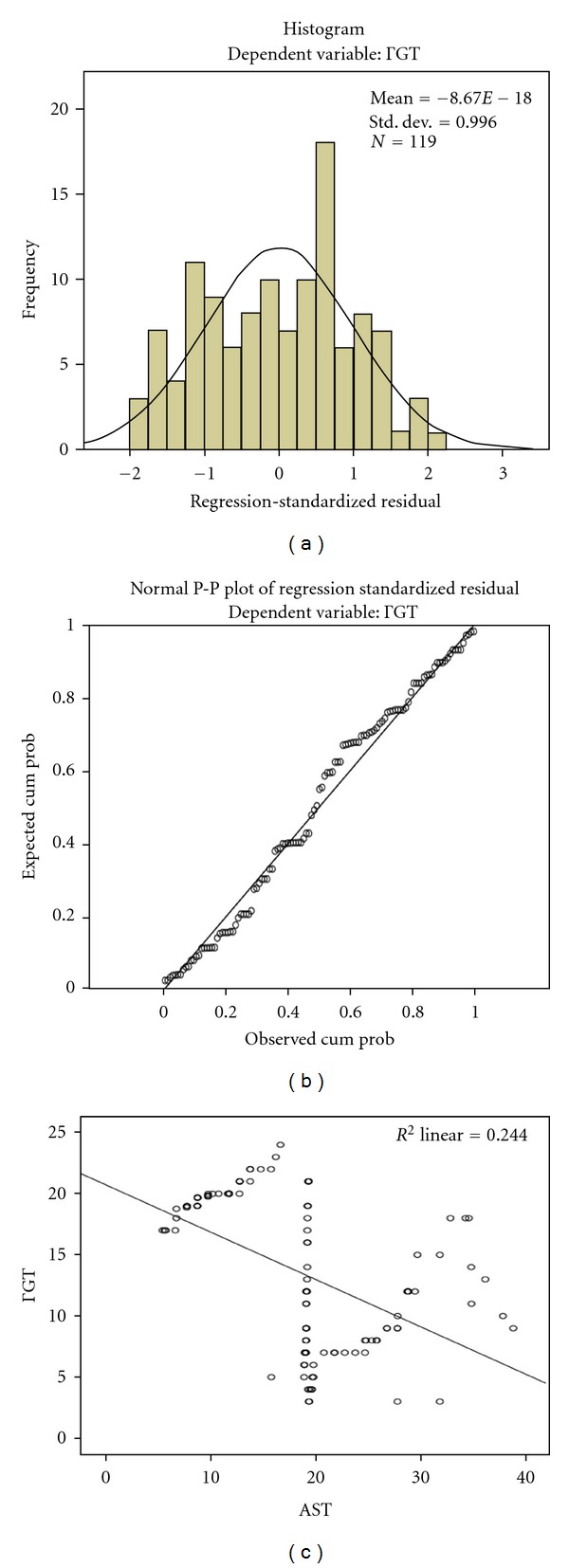
Frequency distribution by histogram (a) and normal *P*-*P* plot of regression-standardized residual (b) for GGT as dependent variable and AST as the only independent determinant (c) during day 0–day 60 anti-TB therapy of initial phase.

Within day 0–day 60, average values of 19.2 ± 7.8 UI/L, 14.8 ± 8.9 UI/L, and 13.3 ± 6.1 UI/L were reported for AST, ALT, and GGT, respectively. There was a negative and significant correlation between day 0–day 60 AST values (*r* = −0.494; *P* < 0.0001) ALT values (*r* = −0.472; *P* < 0.0001), and GGT, respectively. However, a positive and very significant association was noticed between day 0–day 60 AST values (*r* = 0.893; *P* < 0.0001) and day 0–day 60 ALT values. After adjusting for age, ALT, and sex, 23.8% of variations of day 0–day 60 GGT values towards decrease were explained by the increase in day 0–day 60 AST values (standard error = 0.063; *P* < 0.0001) as follows: Y (GGT) = 20.8–0.494 AST.  Figure 3 describes the curves for histogram and normal *P*-*P* plot with day 0–day 60 GGT values as a dependent variable and a reflection of the impact of the initial phase anti-TB therapy: a multivariate and negative association between GGT and AST. 

## 4. Discussion

In this study, the incidence of ADR was only 3%, accidental, transient, benign and due to self-administration of cotrimoxazole. This rate of elevation of hepatic transaminases was within the traditional interval [7], but lower than the levels of 50–75% ADR reported in Eastern European and Asian countries [17]. ADR occurred within the first 30 days of the initiation of anti-TB treatment as usually reported by different studies [17, 18]. Concomitant use of cotrimoxazole or other hepatotoxic drugs is well established as a risk factor for anti-TB-induced liver injury [7, 9, 11, 12].

A significant downward trend in GGT levels was observed from the starting to the end of the initial phase of DOTS. This is because the initial two-month therapy leads to significant improvement in oxidative stress and suppression of inflammation determined by active PTB [2, 3]. Aging, already associated with oxidative stress [19], was one of the exclusion criteria in this study because elderly patients are at a greater risk of oxidative stress and of hepatotoxicity probably because of immunity mechanisms.

In PTB, neutrophils, monocytes, and macrophages are, mobilized to destroy Mycobacteria and generate huge amounts of ROS and lipid peroxidation [20–22]. A series of data obtained from black and white men suggest that serum GGT within its normal range might be an early marker of oxidative stress [13]. The variations of serum GGT in this study, except in one patient with benign ADR, were within their normal range before and after DOTS. The deficiency of antioxidants renders PTB patients unable to cope with their increased oxidative stress [21].

With progressive DOTS during the two months of the initial phase, the baseline relationship between GGT and AST, as well between GGT and ALT changed, respectively. The relationship between GGT and ALT become not significant in considering their average values of day 0–day 60. This means that the anti-TB might neutralise the baseline (day 0, day 30, and day 60) synergistic action of GGT and ALT, both markers of oxidative stress [15]. However, in considering the day 0–day 60 average of liver enzymes, only AST was negatively, significantly and independently associated with serum GGT in these black PTB patients.

## 5. Clinical Implications

The present study will have significant implications in understanding the role of oxidative stress biomarkers and that of liver enzymes in personalizing the DOTS. Systematic steps for prevention of hepatotoxicity are recommended in anti-TB therapy. These recommendations include patient and regimen selection to optimise benefits over risks. Education of patients to avoid self-administration of medications interfering with anti-TB drugs is mandatory.

During the first two months of anti-TB therapy, ALT, AST, and GGT monitoring is recommended. If serum GGT is a marker of oxidative stress targeted by anti-TB during the initial phase, it might have important implications both clinically and epidemiologically because measurement of serum GGT is easy, reliable, and not expensive. It is important to evaluate in tuberculosis patients on DOTS intake of fruits and vegetables, which are rich in antioxidants. Particular attention must be paid in elderly patients at a greater risk of toxicity because of potential poor antioxidant mechanisms [3, 23].

The oxidative stress due to PTB and aggravated by anti-TB therapy hepatotoxicity and the severity of PTB could be reduced by adjuvant therapy with dietary phytochemicals and antioxidants [24, 25].

### 5.1. Limitations of the Study

The study was limited to some degree, as monitoring of AST, ALT, and GGT was not performed monthly from the end of the initial phase and the end of the continuation phase.

## 6. Conclusion

This study concludes that oxidative stress markers within normal range before starting anti-TB treatment and considering conditions related to hepatotoxicity in patients admitted for active PTB might predict excellent tolerability of anti-TB drugs and very low rate of ADR.

The decrease in serum GGT, contrasting with the increase in serum AST and ALT, should reflect the effect of anti-TB therapy during the intensive and initial two-month phase of DOTS. Cotrimoxazole induces a transient enhancement of oxidative stress due to tuberculosis itself and anti-TB chemotherapy.

Clinicians should educate patients about risk factors of anti-TB treatment-induced hepatotoxicity as well as they should be vigilant for conditions related to oxidative stress and deficiency of antioxidant systems.

## Figures and Tables

**Table 1:** Simple correlation coefficients *r* between liver enzymes according to the initial phase therapy time.

Initial	GGT	ALT
Phase	*r* coefficient	*r* coefficient
Time	*P* value	*P* value

Day 0		
AST	0.279	0.735
	*P* = 0.041	*P* < 0.0001
ALT	0.353	1
	*P* = 0.013	

Day 30		
AST	0.397	0.964
	*P* = 0.006	*P* < 0.0001
ALT	0.428	1
	*P* = 0.003	

Day 60		
AST	0.687	0.791
	*P* < 0.0001	*P* < 0.0001
ALT	0.382	1
	*P* = 0.0007	

**Table 2:** Multiple linear regression of GGT levels at day 0, day 30, and day 60 after starting initial phase anti-TB therapy.

Initial phase time	Adjusted *R* ^2^	Standardized beta	Standard error	*P* value
Time independent of test variable				
Day 0				
ALT	10.2%	0.353	0.328	0.025
Day 30				
ALT	16.1%	0.428	0.715	0.007
Day 60	51.6%			
AST		1.027	0.134	<0.0001
ALT		−0.430	0.162	0.023
